# Risk of Iron Overload in Obesity and Implications in Metabolic Health

**DOI:** 10.3390/nu13051539

**Published:** 2021-05-02

**Authors:** Aoibhín Moore Heslin, Aisling O’Donnell, Maria Buffini, Anne P. Nugent, Janette Walton, Albert Flynn, Breige A. McNulty

**Affiliations:** 1 UCD Institute of Food & Health, University College Dublin, Belfield, D04 V1W8 Dublin, Ireland; aoibhin.moore-heslin@ucdconnect.ie (A.M.H.); aisling.odonnell1@ucdconnect.ie (A.O.); maria.buffini@ucd.ie (M.B.); A.Nugent@qub.ac.uk (A.P.N.); 2 Institute for Global Food Security, Queens University Belfast, Belfast BT7 1NN, UK; 3 Department of Biological Sciences, Munster Technological University, T12 P928 Cork, Ireland; Janette.Walton@cit.ie; 4 School of Food & Nutritional Sciences, University College Cork, T12 K8AF Cork, Ireland; A.Flynn@ucc.ie

**Keywords:** ferritin, hepcidin, iron overload, body fat, inflammation, metabolic health

## Abstract

Excessive adiposity is associated with several metabolic perturbations including disturbances in iron homeostasis. Increased systemic inflammation in obesity stimulates expression of the iron regulatory hormone hepcidin, which can result in a maldistribution of bodily iron, which may be implicated in metabolic dysfunction. This study aimed to investigate the effect of adiposity and any associated inflammation on iron homeostasis and the potential implications of dysregulated iron metabolism on metabolic health. Analyses are based on a subsample from the cross-sectional Irish National Adult Nutrition Survey (2008–2010) (*n* = 1120). Ferritin status and risk of iron overload were determined based on established WHO ferritin ranges. Participants were classed as having a healthy % body fat or as having overfat or obesity based on age- and gender-specific % body fat ranges as determined by bioelectrical impedance. Biomarkers of iron status were examined in association with measures of body composition, serum adipocytokines and markers of metabolic health. Excessive % body fat was significantly associated with increased serum hepcidin and ferritin and an increased prevalence of severe risk of iron overload amongst males independent of dietary iron intake. Elevated serum ferritin displayed significant positive associations with serum triglycerides and markers of glucose metabolism, with an increased but non-significant presentation of metabolic risk factors amongst participants with overfat and obesity at severe risk of iron overload. Increased adiposity is associated with dysregulations in iron homeostasis, presenting as increased serum hepcidin, elevated serum ferritin and an increased risk of iron overload, with potential implications in impairments in metabolic health.

## 1. Introduction

Iron is an essential mineral, with integral roles in cellular respiration, DNA synthesis and cellular proliferation [1]. Iron facilitates electron transfer and oxygen delivery in oxidation reduction reactions, which, while essential in maintaining normal cell metabolism, can also result in the generation of toxic reactive oxygen species. Iron-induced oxidative stress in metabolic tissues has been proposed to contribute to the development of some metabolic abnormalities including hepatic steatosis and fibrosis [2], impaired glucose metabolism [3] and dyslipidaemia [4].

A finely controlled balance between iron uptake, storage and utilisation is required to maintain iron homeostasis and ensure optimum functioning of metabolic processes. Iron balance is regulated mainly by the peptide hormone hepcidin, the primary regulator of systemic iron homeostasis. Hepcidin controls the absorption and transport of iron via the iron exporter ferroportin, which regulates intestinal absorption, cellular uptake and storage of iron in tissues [5]. Secreted predominantly by hepatocytes, hepcidin synthesis is regulated by two major mechanisms: iron status and inflammatory stimuli. High circulating iron stimulates hepcidin expression, which induces cellular retention of iron, decreases intestinal absorption of iron and lowers concentrations of circulating free iron [6,7]. Inflammation-induced upregulation of hepcidin expression occurs due to the anti-microbial properties of the hormone, whereby it assumes a two-pronged defensive approach through direct anti-microbial action and decreasing iron availability for the pathogen. While beneficial in cases of acute inflammation, sustained systemic inflammation can result in the prolonged elevation of serum hepcidin, leading to decreased intestinal absorption of iron, sequestration of iron in macrophages and metabolic tissues, and decreased iron availability for erythropoiesis [8].

Obesity associated increases in inflammatory signalling have been linked to increased hepatic hepcidin expression, resulting in disturbed iron homeostasis, characterised by elevated serum ferritin [9] and increased body iron stores [10], but a concurrent decrease in serum iron and transferrin saturation. While the liver is the main site of hepcidin production, the adipose tissue itself has also been revealed as a site of hepcidin synthesis, with elevated expression observed in the adipose tissue of individuals with obesity [11]. Excess adiposity contributes to the dysregulation of iron homeostasis, posing the potential of iron-mediated mechanisms of toxicity to contribute to obesity related metabolic disease.

Existing research has indicated that an increased body mass index (BMI) is associated with disturbances in iron metabolism, presenting as elevated hepcidin [12,13] and hyperferrintinemia, as well as decreased serum iron and transferrin saturation [14]. However, BMI does not provide an accurate depiction of the relationship between adipose tissue and iron homeostasis. Relatively few studies have examined the relationship between the percentage and distribution of body fat and body iron stores. There is some evidence to suggest that a higher body fat percentage is associated with significantly elevated levels of serum hepcidin, hyperferritinaemia, decreased transferrin saturation and increased prevalence of anaemia, potentially attributed to the systemic inflammation induced by excess adiposity [15,16]. This study aimed to investigate the relationship between obesity and disturbances in iron homeostasis and potential implications for metabolic health in an adult cohort using a range of measures of body composition, inflammatory biomarkers, and indicators of iron status.

## 2. Materials and Methods

### 2.1. Study Sample

The National Adult Nutrition Survey (NANS) was a cross-sectional food consumption survey of 1500 free-living Irish adults conducted from 2008 to 2010 [17]. A detailed survey methodology is available at www.iuna.net (accessed on 2 February 2021) and an overview of the methods relevant to this study is outlined below. Ethical approval for this study was received from the Human Research Ethics Committee at the University College Dublin, and by the University College Cork Clinical Research Ethics Committee of the Cork Teaching Hospitals (ECM 3 (p) 4 September 2008). Written, informed consent was obtained from participants before commencing this study in accordance with the Declaration of Helsinki. Participants were demographically representative of the Irish population in terms of the urban–rural divide, age group, sex, and social class, according to 2006 census data (Central Statistics Office, Cork, Ireland, 2007). Analyses for this study were conducted based on a sub-sample of 1120 participants aged between 18 and 84 years (50% male) for whom full blood samples were available, with fasting blood samples available for 79% (*n* = 881).

### 2.2. Dietary Assessment and Analysis

Food and beverage intake data were collected using a 4-consecutive-day, semi-weighed food diary (semi-weighed referring to foods weighed wherever possible), which included at least 1 weekend day. An even spread of food record days across different week and weekend days amongst all participants was ensured so as to make the food consumption data representative of the habitual dietary intake of the Irish adult population. Participants were requested to weigh and record all food, beverages and supplements consumed and, where applicable, record recipes, cooking method, brand and details of leftovers. Initial food intake data were analysed using the food composition database WISP© version 3.0 (Tinuviel Software, Anglesey, UK), which uses data from McCance and Widdowson’s ‘The Composition of Foods’ sixth and fifth editions plus supplemental volumes to generate nutrient intake [18,19,20,21,22,23,24,25,26,27]. Adjustments were made to the food composition database to take account of recipes, nutritional supplements, commonly consumed generic Irish foods and new foods on the market. The database generated from the food and beverage intake data comprised 133,050 rows of data. A total of 2552 food codes were consumed during the survey.

### 2.3. Assessment of Haem, Non-Haem and Fortified Iron Content of Foods

The haem content of each meat-containing food (n-710) was assessed by calculating the proportion of meat within each food item and recipe. Then, the percentage of haem iron relative to each specific origin of meat (beef, lamb, pork, chicken or fish) was calculated using previously published haem values [28], with haem iron attributed to 40% of iron derived from animal products. The remaining 60% of iron in all animal product-containing foods was assumed as non-haem [29]. Other iron-containing food items from plant or other non-meat sources were assumed to contain 100% non-haem based on commonly applied assumptions [30]. Foods fortified with iron were identified by the presence of iron in the ingredient list on the food label using data from a brand-level food label survey derived from the Irish National Food Ingredient Database (INFID v4) [31]. The percentage iron provided by fortification was calculated by comparing against the pre-fortification level of iron in an unfortified equivalent food. Mean daily intake of iron per 1000 kcals (mg/10 MJ) was calculated.

### 2.4. Measurements of Body Composition

All body composition and blood pressure measurements were obtained by trained fieldworkers and according to standard operating procedures [17]. Participants were measured in a non-fasting state, after having voided, wearing light clothing and without shoes. Weight and percentage body fat (% BF) were measured in duplicate using a Tanita body composition analyser BC-420MA (Tanita LTD, GB). Measurements were carried out in line with manufacturer’s instructions. % BF was estimated using standard equations based on the bioimpedance itself and participants age, sex and height. Equations are copyrighted by Tanita^®^ and were created through calibration and validation studies [32,33]. Each % BF reading was completed in duplicate, with a third measurement taken if there was a discrepancy between the two measurements of more than +/−1%. Height was measured to the nearest 0.1 cm using the Leicester portable height measure (Chasmores Ltd. London, UK). Waist and hip circumferences were measured in duplicate to the nearest 0.1 cm using a Seca201 non-stretch tape. Participants aged 20–79years were classified as displaying either a either healthy % BF or as having overfat or obesity using established age- and sex-specific body fat ranges [34]. The current % BF ranges are only suitable for categorisation of those aged between 20 and 79 years, meaning that 88% of the study population (*n* = 986) were categorised as having % BF classed within the healthy, overfat or obese ranges.

### 2.5. Biochemical Analysis

#### 2.5.1. Iron Homeostasis

Full blood counts were analysed on the Beckman Coulter Ac•T diff ™ analyser. Total iron-binding capacity (TIBC) was assessed via the colorimetric TIBC method and serum ferritin using an immunoturbidimetric immunoassay on the Randox Daytona clinical bio-analyser (Randox Laboratories, Crumlin, Co. Antrim, UK). Serum hepcidin-25 was measured using a commercial ELISA kit (DRG Instruments, GmbH, Marburg, Germany). All intra- and inter-assay coefficients of variation were <8.8%. Participants were stratified according to body iron stores based on serum ferritin concentrations in accordance with World Health Organisation thresholds, which identified severe risk of iron overload amongst adults as a serum ferritin >200 ng/mL for males and >150 ng/mL for females [35]. Hyperferritinaemia was assessed based on cutoffs established by the HEIRS study, which identified elevated serum ferritin as >300 µg/L in males and >200 µg/L in females [36,37].

#### 2.5.2. Serum Lipids, Adipocytokines and Markers of Glucose Homeostasis

Serum triacylglycerols, total cholesterol, high-density lipoprotein (HDL) and glucose were analysed using the RX Daytona clinical bio-analyser. Cytokines and hormones (TNF-α, interleukin 2 (IL2), IL6, IL10, insulin, leptin and C-peptide) were measured via the biochip array system (Evidence Investigator, Randox Laboratories, Crumlin, Co. Antrim, UK). ELISA kits were used to measure leptin-soluble receptor (R&D Systems, Oxon, UK) and adiponectin (ALPCO Diagnostics kit, Salem, NH, USA). All intra- and inter-assay coefficients of variations were between ≤4.1% and 18.5%.

### 2.6. Assessment of Visceral Adiposity, Adipose Tissue Dysfunction and Lipid Accumulation

Visceral adiposity index (VAI) was calculated for 841 participants (414 men, 427 women) who provided fasting blood samples and for whom full anthropometric measurements were available. VAI was calculated using an established formula [38] based on measurements of waist circumference (cm), body mass index (kg/m^2^), fasting triglycerides (mmol/L) and high-density lipoproteins (mmol/L). Each participant was classified according to the degree of adipose tissue dysfunction as indicated by VAI according to established age stratified cut-off points [38]. Degree of lipid accumulation was assessed using the lipid accumulation product (LAP) index which is calculated using measurements of waist circumference and fasting serum triglycerides as described elsewhere [39,40].

### 2.7. Assessment of Metabolic Health

Participants were diagnosed with the metabolic syndrome based on the presence of three or more of the metabolic syndrome risk factors defined by the National Cholesterol Education Programme’s Adult Treatment Panel III criteria for metabolic syndrome 2001 [41]. Those missing one or more of the risk factors and those for whom a fasting blood sample was unavailable were excluded, leaving a total of 841 participants (414 males, 427 females) included in this analysis.

### 2.8. Statistical Analysis

All statistical procedures were performed using the Statistical Package for Social Sciences (SPSS version 24.0, Chicago, IL, USA) and all graphs created using Prism version 5 (GraphPad Software Inc). Normality of the distribution of variables was assessed using the Kolmogorov–Smirnov test of normality and through visual inspection of histograms and normal Q–Q plots. Non-normally distributed variables were transformed prior to analysis using the log10 transformation. Due to non-normal distribution all data are presented as the median and interquartile range. Covariate-adjusted general linear model with Bonferroni post hoc was used to compare differences between % BF groups. Covariates used in the model included age, gender, social class, contraception usage, medication use, smoking status, fasting state, frequency of alcohol intake and, for dietary data, under-reporting, as identified using the Goldberg method [42]. Pearson’s chi squared test was applied to categorical data. Analyses were stratified by WHO iron status category as determined by serum ferritin or by % BF category. Linear regression analysis adjusted for age, gender, smoking status, fasting state, medication use, contraception use and frequency of alcohol intake was used to assess the associations between the outcome variables of log-transformed serum ferritin and log-transformed serum hepcidin with anthropometric and biochemical predictors and to analyse relationships between serum ferritin and parameters of metabolic health. Bonferroni correction for multiple comparisons was applied by multiplying each *p* value by the number of traits in each table, with those that exceeded 1.0 marked down to 1.000. Results were deemed statistically significant at *p* < 0.05. Effect sizes are presented for statistically significant results as partial eta squared (ηp^2^). Statistical significance is presented as * in all figures and linear regression analysis, with * *p* < 0.05, ** *p* < 0.01, *** *p* < 0.001.

## 3. Results

### 3.1. Iron Store Status in Obesity

**Figure 1:**
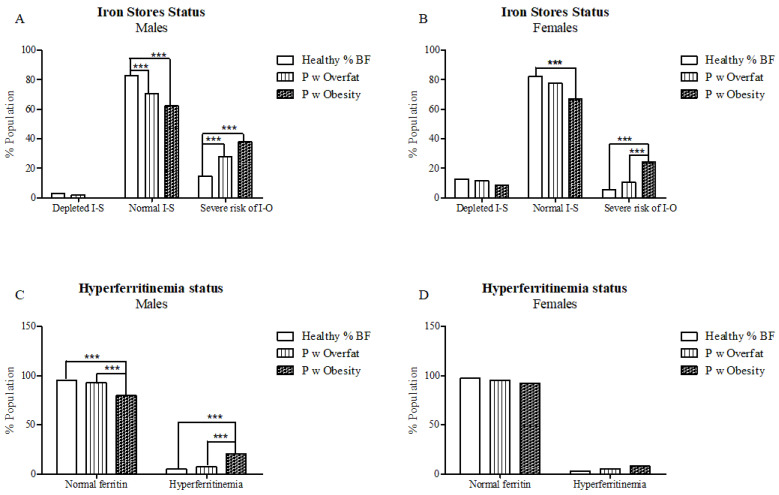
The relationship between % body fat category and (**A**,**B**) WHO iron status category as determined by ferritin and (**C**,**D**) HEIRS ferritin status. *** *p* < 0.001 (Bonferroni correction). X^2^ test of categorical variables, with data presented as % study population. % BF = percentage body fat. P w overfat/obesity = people with overfat/obesity, I-S = iron stores, and I-O = iron overload.

Overall, 41% of participants were classified as having a percentage body fat (% BF) within the healthy range, 35% were within the overfat range, while 24% of participants were classed with obesity, with a greater proportion of overall males deemed as having overfat or obesity (62%) based on age- and gender-specific % BF cutoffs compared to females (55%) (*p* = 0.031) Assessment of iron store status using established WHO ferritin ranges identified 18.0% of the total study population as being at severe risk of iron overload, 76% as having normal iron stores and 6.2% as having depleted iron stores. Prevalence of those at severe risk of iron overload was significantly increased amongst those with overfat and obesity, with 32% of overall participants with obesity and 20% of participants with overfat and were deemed to be at severe risk of iron overload based on ferritin concentrations compared to 10% of participants with a healthy % BF (*p* < 0.001). Risk of iron overload was greatest amongst males with increased %BF, with 28% of males with overfat and 38% with obesity classified as being at severe risk of iron overload (Figure 1A) compared to 11% and 24% of females, respectively (*p* < 0.001) (Figure 1B). Hyperferritinaemia was present in 14.8% of overall participants with obesity compared to 6.2% of participants with overfat and 3.7% of participants with a healthy % BF (*p* < 0.001), with hyperferritinaemia most prevalent amongst males with obesity (21%) (Figure 1C).

Participants deemed to be at severe risk of iron overload according to measures of serum ferritin were on average older, with a higher BMI and % BF and displayed an increased prevalence of adipose tissue dysfunction as determined by visceral adiposity index score (Table 1). % body fat was significantly associated with severe risk of iron overload in males only, with visceral adiposity was significantly associated with severe risk of iron overload in females only (Table 1).

Increasing % body fat was associated with significant increases in serum ferritin and hepcidin amongst males only, with a corresponding significant decrease in hepcidin:ferritin ratio observed amongst males with obesity (Table 2). Hepcidin values for the total population ranged from 0.62 to 31.51 ng/mL, with a median hepcidin value for the total population of 6.52 ng/mL. Overall, males displayed significantly higher median values of serum hepcidin (7.23 ng/mL ± 5.1) compared to females (5.74 ng/mL ± 4.6) (*p* < 0.001), with males classed as having obesity based on % BF displaying the highest values of serum hepcidin (Table 2). Participants classed as having depleted iron stores displayed significantly lower serum hepcidin values (3.09 ng/mL ± 2.9) compared to those at severe risk of iron overload (9.83 ng/mL ± 5.7) (*p* < 0.001). Iron status as indicated by haemoglobin, total iron-binding capacity and mean corpuscular volume generally displayed no or weak associations with increasing % BF, with no differences observed in the prevalence of low haemoglobin stores as determined by WHO cutoffs across categories of % BF (6% people with healthy %BF, 5% people with overfat and 4% people with obesity). Mean daily intakes of dietary iron were similar across all categories of % BF, with no significant differences observed between groups (Supplementary Table S1).

### 3.2. Associations between Measures of Body Composition, Iron Biomarkers and Adipocytokines with Serum Hepcidin and Serum Ferritin

Covariate-adjusted linear regression analysis revealed that % BF was the strongest body composition related predictor of serum hepcidin in males (β = 0.195, *p* < 0.001) (Table 3). Body composition related predictors (BMI, waist circumference and % body fat) displayed significant positive relationships with serum hepcidin and serum ferritin in males only. Serum markers of inflammation exhibited different associations with serum hepcidin between genders, with pro-inflammatory cytokines IL6 and TNFα displaying the strongest relationship in females only. There was no association between inflammatory marker hsCRP and serum hepcidin or serum ferritin observed in either males or females. Serum ferritin was the strongest overall predictor of serum hepcidin and vice versa (Table 3), with a strong inverse relationship observed between serum ferritin and hepcidin:ferritin ratio as expected. % BF displayed the strongest positive relationship with serum ferritin of all body composition variables in males (β = 0.228, *p* < 0.001) only, with only the indexes of central obesity, the VAI and the LAP, exhibiting associations with serum ferritin in females.

### 3.3. Relationship between Elevated Serum Ferritin and Metabolic Health

Covariate-adjusted linear regression analysis investigating the relationship between serum ferritin with blood lipids and gluco-regulatory markers revealed significant positive associations between elevated serum ferritin and increased serum insulin and insulin resistance as indicated by a higher homeostatic model of insulin resistance score and decreased quantitative insulin sensitivity check index score (Table 4).

**Figure 2:**
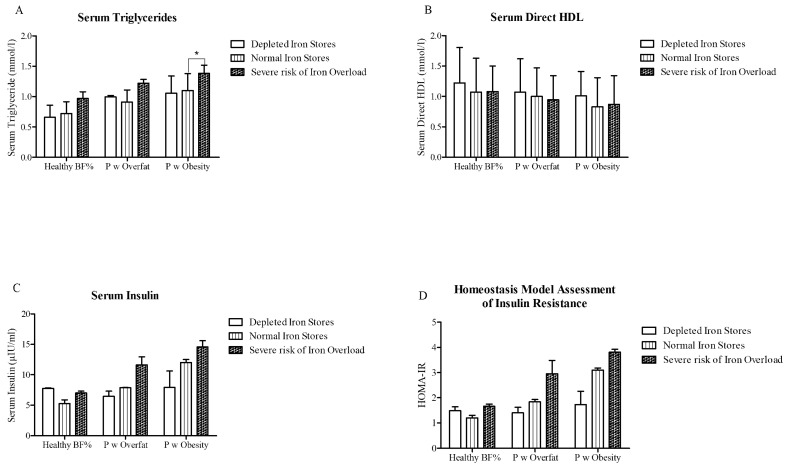
Indices of metabolic health between iron status groups across all categories of BF %: (**A**) serum triglycerides (mmol/L), (**B**) serum direct HDL cholesterol (mmol/L), (**C**) serum insulin (µIU/mL), and (**D**) Homeostasis Model Assessment of Insulin Resistance (HOMA-IR).* *p* < 0.05. ANCOVA of log10-transformed variables (covariates = age, gender, contraception use, and smoking status). Data are presented as the median and IOR. BF % = body fat percentage. P w overfat/obesity = people with overfat/obesity, I-S = iron stores, and I-O = iron overload.

Serum triglycerides displayed a significant positive relationship with serum ferritin, with stratification of the population according to iron status across categories of % BF depicting significantly elevated serum triglycerides amongst participants with obesity at severe risk of iron overload compared to those with obesity and normal iron stores (Figure 2). Iron overload was associated with non-significant increases in serum insulin and HOMA-IR score, and lower HDL cholesterol compared to those with normal or depleted iron stores within the same % BF group (Figure 2).

When examined in relation to metabolic risk, those at severe risk of iron overload had a higher prevalence of displaying 1–2 and >3 risk factors combined across each category of % BF (Table 5). Individuals with obesity who were deemed to be at severe risk of iron overload made up the greatest proportion of participants diagnosed with the metabolic syndrome (39.3%) based on the presence of three or more metabolic risk factors. However, no statistically significant differences between groups were observed. Median serum ferritin was significantly elevated amongst those presenting with three or more metabolic syndrome criteria (121.32 ng/mL ± 134.7) compared to those with no risk factors present (75.29 ng/mL ± 90.6) (*p* = 0.002) for the total population, with males with three or more metabolic risk factors displaying the highest serum ferritin (172.77 ng/mL ± 141.0).

## 4. Discussion

This study describes the iron status of a cohort of individuals classified as having either a healthy % BF or as having overfat or obesity using iron and inflammation associated biomarkers and investigates relationships between disturbed iron homeostasis and metabolic health. Results indicate that a higher % BF amongst males is associated with disturbances in iron homeostasis, presenting as elevated serum hepcidin and serum ferritin, with an increased prevalence of those at severe risk of iron overload. Serum hepcidin levels were elevated in those within the upper categories of % BF, with a related increase in serum ferritin and increased risk of iron overload amongst those with increased body fat.

Existing research has observed significantly elevated serum hepcidin in adults with a higher BMI compared to normal weight counterparts [9,12,43], and loss of fat mass has been shown to normalise serum hepcidin concentrations and improve iron absorption [44]. Few studies have investigated % BF in relation to iron status, with available results indicating a significant relationship between elevated hepcidin and increased total % BF as measured via dual-energy X-ray absorptiometry [45]. Central obesity has also been associated with significantly elevated serum hepcidin in women, with measures of serum hepcidin of 4.48 ng/mL observed in normal weight women compared to 7.85 ng/mL in women with central obesity [16], values that are very much in line with the results of the present study. The serum hepcidin values observed within the present cohort are reflective of certain previously published values, with existing research indicating median hepcidin values of 6.40 ng/mL for a female cohort [45] and a range for values from 3.05 to 37.75 ng/mL for a population of males and females [46]. Considerably higher mean hepcidin values ((19.4 ng/mL ± 10.3) [9] and (88.02 ng/mL) [12]) have been observed amongst those with morbid obesity within bariatric cohorts, with the results of the present study more consistent with the non-obese control groups of these studies. The lower hepcidin values observed within the NANS cohort compared to the bariatric cohorts are likely due to differences in the health status of the population and the low presence of morbid obesity amongst this cohort. Nevertheless, significant increases in serum hepcidin were observed amongst males with excess adipose within this population, with % body fat displaying a significant positive relationship with serum hepcidin. The elevated serum hepcidin observed in individuals with excess adiposity in the current study is likely due, at least in part, to the inflammatory effect of adipose-derived cytokines on the stimulation of hepcidin expression, but also potentially due to the effects of excessive adiposity on hepatic functioning and the relationship between excessive body fat and hepatic steatosis.

Excessive adiposity is associated with an increase in adipose tissue dysfunction and a state of low-grade chronic inflammation stimulated by the secretion of pro-inflammatory adipocytokines from dysfunctional adipose. Those at severe risk of iron overload in this study displayed an increased prevalence of adipose tissue dysfunction (ATD) as indicated by the visceral adiposity index (VAI) in the present study. Adipocytokines produced by dysfunctional adipose tissue trigger hepcidin expression, with pro-inflammatory cytokine IL6 shown to exert a particularly potent effect on hepatic hepcidin expression through direct effect on gene transcription via the IL6–hepcidin axis [47]. IL6 is abundantly expressed in adipose tissue [48], with elevated secretion by adipose tissue observed in individuals with obesity [49]. Visceral adipose-derived IL6 is proposed to enter the hepatic portal vein, directly inducing an inflammatory response in the liver result in hepatic hepcidin production.

Leptin has been shown to directly regulate hepatic hepcidin expression, with elevated circulating leptin linked to hepcidin induced decreased intestinal absorption and macrophage iron retention [50]. Leptin and leptin to adiponectin ratio was the strongest adipocytokine predictor of serum hepcidin in males only, an effect which may potentially be attributed to the effect of central obesity and leptin on liver function and subsequent hepatic hepcidin expression. Population based studies indicate an increased prevalence of non-alcoholic fatty liver disease in males with obesity compared to females [51,52], with accumulating evidence suggesting that leptin may contribute to hepatic steatosis through alteration of insulin signalling with hepatocytes, promoting the accumulation of intracellular fatty acids [53]. Leptin derived from centrally accumulated adipose may serve to exacerbate issues of hepatic lipid accumulation and inflammation, thereby increasing hepcidin expression in males.

While a large degree of the elevated hepcidin observed in subjects with excess adiposity is likely due to the inflammatory effect of adipocytokines on hepatocytes, the adipose tissue itself has also been revealed as a site of hepcidin synthesis [11]. Furthermore, adipose tissue of individuals with obesity has been shown to display enhanced expression of hepcidin [11]. Hepatic hepcidin expression is subject to a feedback control mechanism relating to low transferrin saturation. However, adipocyte hepcidin expression does not appear to be controlled in such a way, with expression instead only correlated with indices of inflammation; IL6 and C-reactive protein [11]. Hepatic hepcidin expression is positively associated with increased body iron stores, while adipose tissue hepcidin expression is positively correlated with BMI [11]. Macrophages have also been shown to secrete hepcidin in response to bacterial pathogens and inflammation [54], suggesting that both adipose tissue located macrophages and the adipocytes themselves may contribute to the overall hepcidin pool in individuals with overfat and obesity. However, hepatic hepcidin gene expression has been shown to be ~700-fold greater compared to expression in the abdominal visceral and subcutaneous adipose tissue [12]. Therefore, the majority of elevated hepcidin production in individuals with obesity is likely to be liver derived, synthesised in response to systemic and potentially hepatic inflammation caused by lipid and iron accumulation. Hepatic iron content has been shown to be significantly elevated in individuals with obesity and is strongly associated with elevated serum hepcidin and ferritin, with serum hepcidin proposed as a reliable indicator of hepatic iron content in individuals with obesity [43].

This study observed that excess adiposity was associated with elevated serum ferritin concentrations and an increased prevalence of severe risk of iron overload and hyperferritinaemia, with a significantly greater prevalence in males than females. Physiological differences play a role in differences in iron status between genders, with pre-menopausal women usually exhibiting lower iron stores due to menstruation. Additionally, hepatic hepcidin synthesis and hepatic inflammation are proposed to be subject to regulation by sex hormones [55], with existing research indicating similarly decreased levels of serum hepcidin amongst healthy females compared to males [56]. There was a greater proportion of males classed as having overfat or obesity based on % BF within this population at 62% of males compared to 55% of females, with males exhibiting significantly higher measures of waist circumference. The increased rates of overfat and obesity and measures of central obesity amongst males may contribute to the elevated hepcidin, ferritin and risk of iron overload observed compared to females. Obesity has been associated with disturbed iron homeostasis, presenting as “dysmetabolic iron overload syndrome” (DIOS), with hyperferritinaemia observed amongst those with obesity [14,57]. Increased liver and body iron stores are a feature of DIOS, with this iron overload not due to any identifiable cause such as excessive dietary iron consumption [58]. This is in line with results of the present study, with intake of dietary iron not implicated in the iron overload observed in those with obesity.

The chronic, low-grade systemic inflammation of obesity stimulates both the expression of hepcidin and an increase in serum ferritin levels, as ferritin is an acute-phase protein and is affected by the presence of inflammation. While this study observed no associations between inflammatory biomarker hsCRP with serum ferritin, the systemic inflammation of obesity is likely to contribute to the increase in serum ferritin observed amongst those with overfat and obesity in this population. Elevated levels of circulating ferritin have been significantly associated with adverse health outcomes following adjustment for inflammation, with a meta-analysis proposing the possibility of a direct causal effect between ferritin and type 2 diabetes [59], with iron-induced oxidative stress proposed as a potentially mediating effect [60,61].

Elevated serum hepcidin induces cellular retention of iron, reducing iron export from enterocytes, macrophages and hepatocytes, resulting in tissue iron overload. Sequestration of iron within metabolic tissues can lead to a proliferation of oxidative damage and impaired metabolic functioning. In this study, individuals with obesity at severe risk of iron overload displayed the highest incidence of severe adipose tissue dysfunction. The association between iron overload with % BF and indices of adipose tissue dysfunction may indicate that ferritin has direct or indirect effects on adiposity. The relationship between inflammation and dysregulated iron homeostasis may be bidirectional, with inflammation both a cause and result of intracellular iron retention. Inflammation-induced iron retention in macrophages provides the intracellular free iron required for activation NF-кB, a promoter of cytokine production. Iron retention in hepatic and adipose tissue may proliferate the issues of systemic inflammation associated with obesity. Levels of hepcidin expression are strongly correlated with the degree of iron deposition in the liver [62], proposing a potential causal role of ferritin in hepatic inflammation and the subsequent stimulation of hepcidin expression.

The link between iron overload and oxidative stress has been associated with obesity associated pathologies including metabolic dysfunction, type 2 diabetes and CVD. In this study, individuals deemed to be at severe risk of iron overload displayed a more unfavourable metabolic profile and increased prevalence of metabolic risks factors compared to those of a similar % BF with depleted or normal iron stores. The results of this study indicate that elevated serum ferritin may be associated with a trend towards impaired glucose homeostasis, with serum ferritin displaying significantly positive relationships with serum insulin and HOMA-IR and an inverse relationship with QUICKI. Serum ferritin levels have been positively associated with degree of insulin resistance and an increased presentation of components of the metabolic syndrome [63]. Accumulating evidence suggests a link between bodily iron excess and features of insulin resistance [64] and the metabolic syndrome presenting as elevated triglycerides, low-density lipoprotein cholesterol, insulin, HOMA-IR and hypertension [65]. Serum ferritin exerts a lipolytic effect on adipocytes [66] resulting in excess circulating free fatty acids which contribute to insulin resistance [67]. DIOS has been shown to predict the onset of type 2 diabetes mellitus and non-alcohol fatty liver disease [68]. The adverse effects of iron overload on metabolic health are reflective of symptoms observed in those with hemochromatosis, which is associated with impaired functioning of the liver, pancreas, heart and endocrine organs due to iron-induced cellular damage [69]. Reduced plasma ferritin concentrations have been associated with a decreased risk of type 2 diabetes mellitus [70] and improvements in lipid profile [71], indicating that consideration of iron status is an important factor in the treatment of obesity associated metabolic dysfunction.

This study benefited from the large, nationally representative sample size of the NANS population, for which a wide variety of body composition measures and biochemical data are available. The use of bioelectrical impedance analysis determined % BF measurements provides a unique insight into the relationship between adiposity and iron homeostasis which, when used in conjunction with biomarkers of systemic inflammation, may indicate potential mechanisms related to the elevated hepcidin and hyperferritineamia observed in the population. This is the first study investigating the relationship between iron metabolism and obesity and the possible related impacts on metabolic health within a nationally representative cohort of Irish adults. The detailed dietary records of the National Adult Nutrition Study facilitated the analysis of dietary iron intake, a measure which has been relatively lacking in existing studies pertaining to iron homeostasis within obesity. This study could have been strengthened through the investigation of other markers of iron status such as serum transferrin receptor (sTFr), an iron status indicator that is not affected by inflammation. Measures of serum sTFr were not available for this population and may have been useful in accurately determining the effects of inflammation on iron status. Serum iron biomarkers were used in the assessment iron status and risk of iron overload in this study, which, while useful in providing an indication of risk, do not provide a true assessment of iron overload. Serum ferritin is an acute-phase reactant and has been found to rise sharply with inflammation [72]. While no association between serum ferritin and the inflammatory marker hsCRP was observed in this study, it is possible that measures of serum ferritin may have been influenced by inflammation. The use of magnetic resonance imaging in the assessment of bodily iron status, particularly with regard to hepatic iron content, would provide insight into total body iron burden and a true measure of iron overload. However, due to the population-based nature of the NANS study, such assessments are unfeasible. In this study, indices of visceral adiposity and anthropometric measures were used to provide estimates of visceral obesity; however, these indices do not provide a true measure of visceral adipose tissue. The use of bioelectrical impedance analysis as a means of assessing % body fat is a convenient and safe method for assessing body composition and has been proposed as appropriate for use in large-scale epidemiological studies. However, bioelectrical impedance does carry the risk of over or under estimation of fat mass [73] and is not the gold-standard method compared to dual-x-ray absorptiometry, a method which was unfeasible within the NANS study.

This study indicates the potential relationships between body fat-associated inflammation and iron status, with the inflammatory effects of excessive adiposity potentially contributing to elevated serum hepcidin and disturbed iron homeostasis. Dysregulation of iron metabolism due to elevated hepcidin expression and increased ferritin increases the severe risk of iron overload, which may be implicated in metabolic dysfunction and increased risk of metabolic disease. Further research investigating the molecular mechanisms linking adipose-associated inflammation to dysmetabolic iron overload and its potential role in the pathogenesis of metabolic disease is warranted.

## Figures and Tables

**Table 1:** Age and body composition characteristics of the population across categories of iron store status as determined by WHO serum ferritin range.

	Depleted Iron Stores (*n* = 69)		Normal Iron Stores (*n* = 849)		Severe Risk of Iron Overload (*n* = 202)			
	Median	IQR	Median	IQR	Median	IQR	*p* ^†^	ηp^2^
Age (years)								
Total	43.00 ^a^	15	39.00 ^a^	26	52.00 ^b^	21	<0.001	0.051
Male	53.00 ^a,b^	25	36.00 ^a^	25	48.00 ^b^	23	<0.001	0.042
Female	42.50 ^a^	14	41.00 ^a^	26	57.00 ^b^	16.5	<0.001	0.063
Body mass index (kg/m^2^)								
Total	25.42 ^a,b^	5.21	25.81 ^a^	5.82	28.82 ^b^	5.65	<0.001	0.031
Male	27.06 ^a,b^	3.54	26.61 ^a^	5.17	28.81 ^b^	5.16	<0.001	0.032
Female	25.14 ^a,b^	5.57	25.25 ^a^	5.8	29.04 ^b^	6.93	0.032	0.023
Waist circumference (cm)								
Total	83.15 ^a,b^	14.86	88.50 ^a^	19.7	99.50 ^b^	17.7	0.035	0.011
Male	92.2	15.8	93.63	18	101.05	16.2	0.368	-
Female	82.5	13.98	84.05	17	94.05	18.23	1.00	-
Waist:hip ratio								
Total	0.84	0.11	0.87	0.13	0.92	0.11	0.480	-
Male	0.9	0.1	0.9	0.12	0.95	0.1	0.400	-
Female	0.82	0.11	0.84	0.11	0.86	0.11	1.00	-
% Body fat								
Total	32.70 ^a,b^	9.5	27.88 ^a^	13.38	29.28 ^b^	14.59	<0.001	0.025
Male	22.10 ^a,b^	8.63	22.05 ^a^	10.35	26.55 ^b^	7.9	0.008	0.028
Female	33.7	7.55	34.25	10.5	40.4	9.13	0.400	-
Lipid accumulation product index								
Total	23.06 ^a,b^	25.19	26.95 ^b^	31.12	46.46 ^b^	45.62	<0.001	0.022
Male	38.61	61.82	32.15	33.37	44.25	51.53	0.440	-
Female	22.47	24.57	24.31	24.47	48.36	46.26	0.064	-
Visceral adiposity index								
Total	0.96 ^a^	0.67	1.06 ^a^	0.87	1.39 ^b^	1.28	<0.001	0.025
Male	1.29	1.34	1.09	0.97	1.24	1.11	0.712	-
Female	0.09 ^a^	0.62	1.05 ^a^	0.7	1.45 ^b^	1.46	<0.001	0.044
% population displaying adipose tissue dysfunction								
Total	7.8% ^a^		12.7% ^a^		25% ^b^		<0.001	
Male	14.30%		13.90%		21.60%		1.00	
Female	6.8% ^a^		11.6% ^a^		31.9% ^b^		<0.001	

ANCOVA for continuous variables; X^2^ test for categorical variables; covariates = age, social class, gender, smoking status, contraception use, medication use, and frequency of alcohol intake. *p* = overall *p* values indicate significant differences between groups by ANCOVA/X^2^ test. *p* < 0.05, Bonferroni correction for multiple comparisons. Statistically significant differences between groups are indicated by alternating superscript letters. ^†^ = log10-transformed variable. Iron store status determined based on WHO serum ferritin cutoffs. (*n* = 1120—total population).

**Table 2:** Iron biomarkers across categories of % body fat.

	People with Healthy % BF (*n* = 407)		People with Overfat (*n* = 342)		People with Obesity (*n* = 237)			
	Median	IQR	Median	IQR	Median	IQR	*p* ^†^	ηp^2^
Serum Hepcidin (ng/mL)								
Total	5.62 ^a^	4.46	6.78 ^b^	4.91	7.73 ^b^	5.1	<0.001	0.017
Male	6.45 ^a^	4.42	7.49 ^ab^	5.28	9.06 ^b^	4.67	0.048	0.021
Female	4.94	4.16	5.76	4.86	7.21	4.83	0.270	-
Serum Ferritin (ng/mL)								
Total	75.91 ^a^	93.94	98.65 ^b^	120.63	132.86 ^b^	137.23	<0.001	0.026
Male	120.50 ^a^	91.93	149.68 ^a^	112.52	175.91 ^b^	138.04	<0.001	0.051
Female	39.69	52.46	70.27	84.00	67.55	116.31	0.984	-
Hepcidin:Ferritin Ratio								
Total	0.08	0.08	0.07	0.06	0.06	0.06	0.216	-
Male	0.06 ^a^	0.05	0.05 ^a,b^	0.04	0.048 ^b^	0.04	0.042	0.021
Female	0.10	0.10	0.09	0.08	0.10	0.08	1.00	-
Haemoglobin (g/dL)								
Total	14.10 ^a^	2.00	14.20 ^a,b^	1.90	14.50 ^b^	1.83	0.048	0.01
Male	15.10	1.50	15.10	1.40	15.30	1.4	0.066	-
Female	13.30	1.10	13.50	1.30	13.50	1.45	1.00	-
Mean Corpuscular Volume (fL)								
Total	91.20	5.10	90.50	5.00	90.30	5.2	0.132	-
Male	90.80	5.10	90.65	4.88	90.90	4.95	1.00	-
Female	91.55 ^a^	5.07	90.20 ^a,b^	5.30	89.90 ^b^	5.35	0.03	0.024
Serum Total Iron-Binding Capacity (TIBC) (µmol/L)								
Total	59.15	11.71	58.96	11.53	60.30	9.38	1.00	-
Male	56.84	8.16	58.20	10.32	59.10	11.3	1.00	-
Female	61.60	13.41	59.87	11.02	61.70	11.74	1.00	-

ANCOVA comparing iron biomarkers across categories of % body fat; covariates = age, social class, gender, smoking status, fasting state, medication use, contraception use, and frequency of alcohol intake. *p* = overall *p* values indicate significant differences between groups by ANCOVA. ^†^ = log10-transformed variable. *p* < 0.05 (Bonferroni adjustment for multiple comparisons); ηp2—partial eta squared effect size. Statistically significant differences between groups are indicated by alternating superscript letters. (*n* = 986—includes adults aged 20–79 to whom existing % BF ranges apply).

**Table 3:** Adjusted linear regression analysis for predictors of serum hepcidin and serum ferritin.

	Serum Hepcidin †		Serum Ferritin †	
	Males (β (95% CI))	Females (β (95% CI))	Males (β (95% CI))	Females (β (95% CI))
Body composition				
BMI (kg/m2) ^†^	0.175 (0.31, 0.93) ***	0.097 (0.04, 0.66)	0.189 (0.51, 1.40) ***	0.068 (−0.08, 0.75)
% Body fat ^†^	0.195 (0.15, 0.47) ***	0.067 (−0.07, 0.45)	0.228 (0.29, 0.74) ***	0.064 (−0.10, 0.59)
Waist circumference (cm) ^†^	0.158 (0.24, 1.06) *	0.065 (−0.10, 0.69)	0.187 (0.50, 1.65) ***	0.058 (−0.17, 0.87)
Waist:hip ratio ^†^	0.149 (0.29, 1.55)	0.046 (−0.29, 0.94)	0.160 (0.49, 2.27) *	0.047 (−0.37, 1.26)
Visceral adiposity index ^†^	0.067 (−0.03, 0.15)	0.084 (−0.01, 0.20)	0.146 (0.06, 0.31)	0.198 (0.17, 0.44) ***
Lipid accumulation product ^†^	0.105 (−0.001, 0.14)	0.097 (0.001, 0.17)	0.178 (0.07, 0.27) *	0.149 (0.06, 0.29) *
Iron biomarkers				
Serum ferritin ^†^	0.509 (0.33, 0.43) ***	0.532 (0.34, 0.46) ***	-	-
Serum hepcidin ^†^	-	-	0.516 (0.59, 0.79) ***	0.521 (0.60, 0.79) ***
Hepcidin:ferritin ratio ^†^	0.246 (0.15, 0.28) ***	0.246 (0.16, 0.30) ***	−0.677 (−0.86,−0.72) ***	−0.630 (−0.85, −0.70) ***
Adipocytokines				
Serum IL6 ^†^	0.055 (−0.02, 0.10)	0.198 (0.09, 0.22) ***	−0.027 (−0.12, 0.06)	0.145 (0.06, 0.24) *
IL6:IL10 ratio ^†^	0.041 (−0.03, 0.09)	0.110 (0.01, 0.15)	−0.003 (−0.09, 0.08)	0.111 (0.02, 0.20)
Serum TNFα ^†^	0.036 (−0.08, 0.22)	0.136 (0.12, 0.48) *	−0.029 (−0.29, 0.14)	0.112 (0.09, 0.57)
Serum Hs CRP ^†^	0.102 (0.005, 0.13)	0.085 (−0.01, 0.12)	0.006 (−0.08, 0.09)	0.051 (−0.04, 0.14)
Serum leptin ^†^	0.126 (0.02, 0.18)	0.008 (−0.06, 0.07)	0.181 (0.09, 0.33) *	0.025 (−0.06, 0.11)
Serum adiponectin ^†^	−0.124 (−0.27, −0.06) *	−0.052 (−0.19, 0.04)	−0.136 (−0.41, −0.11) *	−0.043 (−0.24, 0.07)
Leptin: adiponectin ratio ^†^	0.158 (0.04, 0.17) *	0.031 (−0.03, 0.07)	0.238 (0.13, 0.32) ***	0.042 (−0.03, 0.10)

Covariate-adjusted linear regression analysis. Covariates = age, smoking status, fasting state, contraception use, medication use, and frequency of alcohol intake. ^†^ = log10-transformed variable. β = standardised beta coefficient (95% confidence interval); *p* < 0.05 (Bonferroni adjustment for multiple comparisons). Statistically significant results are indicated by *, with * *p* < 0.05 and *** *p* < 0.001. BMI—body mass index, IL6—interleukin 6, and Hs CRP—high-sensitivity C reactive protein. (*n* = 1120—total population)

**Table 4:** Adjusted linear regression analysis of relationship between serum ferritin and metabolic health parameters.

Serum Ferritin						
	Model 1		Model 2		Model 3	
	β (95% CI)	*p* ^†^	β (95% CI)	*p* ^†^	β (95% CI)	*p* ^†^
Total cholesterol	0.064 (0.00, 0.03)	0.248	0.04 (−0.01, 0.02)	1.00	0.044 (−0.05, 0.03)	1.00
HDL cholesterol	−0.231 (−0.08,−0.05)	<0.001	−0.267 (−0.09, −0.06)	<0.001	−0.06 (−0.03, 0.001)	0.496
LDL cholesterol	0.082 (0.01, 0.05)	0.048	0.07 (0.003, 0.05)	0.232	0.015 (−0.02, 0.03)	1.00
Triglycerides	0.272 (0.11, 0.17)	<0.001	0.289 (0.12, 0.18)	<0.001	0.183 (0.06, 0.13)	<0.001
Insulin	0.137 (0.07, 0.17)	<0.001	0.187 (0.11, 0.21)	<0.001	0.087 (0.02, 0.12)	0.040
Glucose	0.209 (0.03, 0.05)	<0.001	0.180 (0.02, 0.04)	<0.001	0.092 (0.004, 0.03)	0.072
HOMA-IR	0.165 (0.10, 0.21)	<0.001	0.204 (0.13, 0.24)	<0.001	0.099 (0.04, 0.15)	0.012
QUICKI	−0.160 (−0.03, −0.01)	<0.001	−0.203 (−0.04, −0.02)	<0.001	−0.094 (−0.02, −0.004)	0.024

Linear regression analysis of associations between serum ferritin and parameters of metabolic health. Model 1—unadjusted Model 2—adjusted for age and % body fat, Model 3—adjusted for age, % body fat, gender, smoking status, fasting state, medication use, fasting state, and frequency of alcohol intake. *p* < 0.05 (Bonferroni adjustment for multiple comparisons). HOMA—Homeostasis Model Assessment; QUICKI—quantitative insulin sensitivity check index. *n* = 1120—total population; ^†^ = log10-transformed variable.

**Table 5:** Risk of metabolic syndrome between iron status groups across categories of % BF.

	People with Healthy % BF				People with Overfat				People with Obesity			
	Depleted Iron Stores	Normal Iron Stores	Severe Risk of Iron Overload	*p*	Depleted Iron Stores	Normal Iron Stores	Severe Risk of Iron Overload	*p*	Depleted Iron Stores	Normal Iron Stores	Severe Risk of Iron Overload	*p*
No Risk Factors	83.30%	64.60%	42.80%	0.059	41.20%	35.30%	27.50%	0.527	0.00%	6.60%	7.10%	0.223
1–2 Risk Factors	16.70%	34.30%	53.60%		52.90%	52.50%	64.70%		75.00%	70.40%	53.60%	
≥3 Risk Factors	0.00%	1.10%	3.60%		5.90%	12.20%	7.80%		25.00%	23.00%	39.30%	

*X*^2^ test of categorical variables: risk of metabolic syndrome determined based on National Cholesterol Education Programme’s Adult Treatment Panel III criteria for metabolic syndrome 2001—participants presenting with three or more risk factors are diagnosed with Metabolic syndrome. Details of diagnosis criteria detailed above. Table includes participants for whom all criteria for cut-off points were available based on analysis of fasted blood samples. (*n* = 853).

## Data Availability

The data presented in this study are available from the corresponding author upon reasonable request.

## Supplementary Materials

The following are available online at https://www.mdpi.com/article/10.3390/nu13051539/s1, Table S1: Mean daily dietary iron intakes mg/10 MJ across categories of % body fat.
