# Supplementary material for: Risk of Iron Overload in Obesity and Implications in Metabolic Health
Source: Nutrients. 2021 May 2;13(5):1539. doi: 10.3390/nu13051539 (PMC8147503; doi:10.3390/nu13051539)
Supplement: Supplementary file 1 [file nutrients-13-01539-s001.zip › nutrients-1187757-supplementary.pdf]

Supplementary Table S1: Mean daily dietary iron intakes mg/10MJ across categories of % body fat.

	**People with healthy % BF**		**People with overfat**		**People with obesity**			
	Median	IQR	Median	IQR	Median	IQR	*p†*	*np*[2]
**MDI Iron (mg/10MJ)**								
Total	12.40	6.62	12.01	6.68	10.83	5.94	0.61	-
Males	14.55	7.35	13.69	6.96	11.89	5.73	0.17	-
Females	10.96	5.38	10.24	5.09	9.57	4.55	1.00	-
**MDI Haem Iron (mg/10MJ)**								
Total	0.84	0.91	0.89	0.88	0.92	1.04	0.44	-
Males	1.10	1.07	1.09	1.00	1.21	1.18	1.00	-
Females	0.58	0.69	0.65	0.76	0.64	0.63	1.00	-
**MDI Non-haem iron (mg/10MJ)**								
Total	9.02	4.56	8.57	4.20	8.13	4.27	1.00	-
Males	10.28	4.24	9.65	4.30	9.71	4.28	1.00	-
Females	8.04	3.54	7.97	3.31	7.22	3.1	1.00	-
**MDI Fortified iron (mg/10MJ)**								
Total	1.59	4.52	1.29	3.89	0.00	2.37	0.80	-
Males	2.21	5.33	1.76	4.55	0.00	2.37	0.11	-
Females	1.06	3.57	1.21	3.41	0.43	2.39	1.00	-
**MDI Fortified Iron from supplements (mg/10MJ)**								
Total	0.00	0.00	0.00	0.00	0.00	0	1.00	-
Males	0.00	0.00	0.00	0.00	0.00	0	1.00	-
Females	0.00	0.00	0.00	0.00	0.00	0	1.00	-
**MDI Fortified Iron foods (mg/10MJ)**								
Total	1.07	3.40	0.72	3.03	0.00	2.13	1.00	-
Males	1.68	4.52	1.00	4.10	0.00	2.13	0.46	-
Females	0.77	2.82	0.57	2.62	0.00	2.18	1.00	-

MDI - Mean daily intake of dietary iron calculated as mg per 1000kcal (10MJ) † = Log10 transformed variable, ANCOVA (Covariates = age, gender, under-reporting, social class, smoking status) variables. $p<0.05$ (Bonferroni adjustment for multiple comparisons).
